# Splicing mutations in *AMELX* and *ENAM* cause amelogenesis imperfecta

**DOI:** 10.1186/s12903-023-03508-8

**Published:** 2023-11-20

**Authors:** Zhenwei Zhang, Xiaoying Zou, Lin Feng, Yu Huang, Feng Chen, Kai Sun, Yilin Song, Ping Lv, Xuejun Gao, Yanmei Dong, Hua Tian

**Affiliations:** 1 Department of Cariology and Endodontology, National Clinical Research Center for Oral Diseases, National Engineering Laboratory for Digital and Material Technology of Stomatology, Beijing Key Laboratory of Digital Stomatology, Peking University School and Hospital of Stomatology, No. 22 Zhongguancun Nandajie, Haidian District, Beijing, 100081 PR China; 2 Department of Medical Genetics, School of Basic Medical Sciences, Peking University Health and Science Center, Beijing, PR China; 3 Central Laboratory, National Center of Stomatology, National Clinical Research Center for Oral Diseases, National Engineering Laboratory for Digital and Material Technology of Stomatology, Beijing Key Laboratory of Digital Stomatology, Peking University School and Hospital of Stomatology, Beijing, PR China; 4 Department of Prosthodontics, National Clinical Research Center for Oral Diseases, National Engineering Laboratory for Digital and Material Technology of Stomatology, Beijing Key Laboratory of Digital Stomatology, Peking University School and Hospital of Stomatology, Beijing, PR China

**Keywords:** Amelogenesis imperfecta, *AMELX*, *ENAM*, Exon skipping, Intron retention

## Abstract

**Background:**

Amelogenesis imperfecta (AI) is a developmental enamel defect affecting the structure of enamel, esthetic appearance, and the tooth masticatory function. Gene mutations are reported to be relevant to AI. However, the mechanism underlying AI caused by different mutations is still unclear. This study aimed to reveal the molecular pathogenesis in AI families with 2 novel pre-mRNA splicing mutations.

**Methods:**

Two Chinese families with AI were recruited. Whole-exome sequencing and Sanger sequencing were performed to identify mutations in candidate genes. Minigene splicing assays were performed to analyze the mutation effects on mRNA splicing alteration. Furthermore, three-dimensional structures of mutant proteins were predicted by AlphaFold2 to evaluate the detrimental effect.

**Results:**

The affected enamel in family 1 was thin, rough, and stained, which was diagnosed as hypoplastic-hypomature AI. Genomic analysis revealed a novel splicing mutation (NM_001142.2: c.570 + 1G > A) in the intron 6 of amelogenin (*AMELX*) gene in family 1, resulting in a partial intron 6 retention effect. The proband in family 2 exhibited a typical hypoplastic AI, and the splicing mutation (NM_031889.2: c.123 + 4 A > G) in the intron 4 of enamelin (*ENAM*) gene was observed in the proband and her father. This mutation led to exon 4 skipping. The predicted structures showed that there were obvious differences in the mutation proteins compared with wild type, leading to impaired function of mutant proteins.

**Conclusions:**

In this study, we identified two new splicing mutations in *AMELX* and *ENAM* genes, which cause hypoplastic-hypomature and hypoplastic AI, respectively. These results expand the spectrum of genes causing AI and broaden our understanding of molecular genetic pathology of enamel formation.

**Supplementary Information:**

The online version contains supplementary material available at 10.1186/s12903-023-03508-8.

## Introduction

Dental enamel is highly mineralized tissue occupied by large hydroxyapatite crystals that are organized into prisms [1]. Enamel formation (amelogenesis) is the result of a complex biomineralization process, which is coordinated reciprocal interactions between ectoderm and mesenchyme [2]. Ameloblasts are differentiated from inner enamel epithelium cells and secrete multiple extracellular matrix proteins into the developing enamel layer [3]. As the crystals ribbons undergo nucleation and elongation, the matrix proteins are cleaved and degraded by proteases and reabsorbed by ameloblasts to allow the mineral ribbons to thicken and widen, finally achieving fully mineralization with remarkable hardness [4]. Any genetic or environmental disturbances can cause developmental enamel defects in a localized or generalized pattern [5].

Amelogenesis imperfecta (AI) is an inherited disorder affecting tooth enamel formation which is genetically and phenotypically heterogeneous [6]. AI exhibit diverse clinical phenotypes depending on the stages of disturbance occurrence [7]. Based on clinical appearance, cases of enamel malformation are categorized as hypoplastic, hypomaturation and hypocalcified types [8]. Identifying the genes that causing isolated AI provide the molecular clues for dental enamel formation. To date, more than 27 genes are known to be involved in the molecular pathogenesis of AI, in which amelogenin (*AMELX*, Xp22.3-Xp22.1), enamelin (*ENAM*, 4q21), ameloblastin (*AMBN*, 4q21), kallikrein related peptidase 4 (*KLK4*, 19q13), matrix metallopeptidase 20 (*MMP20*, 11q22) are common candidate genes reported [5, 9, 10]. The disease-causing mutations are usually characterized as missense, nonsense, or frameshift mutations [11]. Moreover, mutation at exon-intron boundaries can leads to retention of the intron, or exon skipping [12]. To date, around 10% mutations occurred in exon-intron boundary were reported to cause AI, including *AMBN* (1), *CNNM4* (1), *COL17A1* (1), *ENAM* (7), *FAM20A* (8), *LAMB3* (1), *LTBP3* (1), *MMP20* (4), *ODAPH* (1), *RELT* (1), *SLC10A7* (3), and *TP63* (1) [13].

Amelogenin is the most abundant extracellular matrix protein mainly expressed by preameloblasts and ameloblasts, which plays a vital role in hydroxyapatite crystal elongation and growth [2]. *AMELX* has 7 exons and multiple isoforms resulting from conserved alternative splicing in the mRNA transcripts [14]. *AMELX* mutations lead to X-linked AI, which is often manifested as thinner enamel and hypomatured teeth with brown discoloration [15]. At present, more than 28 pathogenic *AMELX* mutations have been reported [16]. It is reported that exon 4 is usually skipped during pre-mRNA splicing and internal splicing sites can be observed in exon 6 [17]. Silent mutation in exon 4 was reported to cause generalized pitted hypoplastic AI by inclusion of exon 4 during transcription process [14]. The phenotypes varied from a deficiency in the thickness (hypoplasia) to mineralization (hypomineralization/hypomaturation) [10]. Characterization in domains of AMELX help to provide the clues to understanding diverse phenotypes.

Enamelin is the largest and accounts for about 5% of the enamel matrix proteins, which is mainly expressed in the secretory ameloblasts and participates in nucleation and extension of enamel crystals during enamel formation [18, 19]. Mutations of *ENAM* lead to hypoplasitc AI by an autosomal dominant or recessive inheritance pattern. Up to now, 24 pathogenic mutations of *ENAM* have been reported [13]. Human enamelin gene contains 10 exons, in which exon 2 is usually skipped during pre-mRNA splicing. Splicing donor site mutation (NM_031889.3: c.-61 + 1G > A) was reported to result in a retention of intron 1 and exon 2, presumably disturbing regulation transcription of 5’UTR of enamelin gene [20]. However, the mechanism of AI caused by splicing mutations is still unclear.

In the current study, we performed a mutational analysis in 2 Chinese families presenting with hypoplastic and hypomaturation AI. We identified two novel splicing mutations in *AMELX* and *ENAM*, respectively. Splicing assay confirmed the effects of pre-mRNA splicing mutation to further reveal the genotype-phenotype correlation with AI causative genes.

## Materials and methods

### Recruitment of families with AI

The protocol of this study was performed in accordance with the Declaration of Helsinki principles and ethically reviewed and approved by the Ethical Committee of Peking University School and Hospital of Stomatology (PKUSSIRB-202,278,104). Written informed consent was obtained from all participants or their guardians. Two Chinese families with AI were recruited for this study. The probands of the two families sought medical advice for dental caries. Clinical and radiological features were obtained. Medical and feeding history in childhood were also collected to exclude environmental induced enamel hypoplasia.

### Whole-exome sequencing

Peripheral venous blood and saliva of family members were collected. Genomic DNA was extracted using a DNA TIANamp Blood DNA mini kit (TIANGEN, Beijing, China) for blood and a MagMAX gDNA Saliva Isolation Kit (Thermofisher, Waltham, MA, USA) for saliva following the manufacturer’s instructions. After amplification, DNA products were sheared to obtain 150 to 200 bp fragments for next library preparation using TruSeq DNA Sample Prep Kit (Illumina, San Diego, CA, USA). Sequencing was conducted using Illumina sequencing platform (Illumina) in the Euler Genomics (Euler, Beijing, China). Candidate genes were filtered as follows (Supplemental Tables 1 and 2). Firstly, the benign or suspected benign mutations and synonymous mutations were excluded from the gene list. Then, single nucleotide variants (SNVs) and insertions/deletions (InDels) with a minor allele frequency (MAF) > 0.01 in bioinformatics databases, including the 1000 Genomes Project data in Ensembl (http://asia.ensembl.org/Homo_sapiens/Info/Index), the Genome Aggregation Database (gnomAD, http://gnomad.broadinstitute.org), the single nucleotide polymorphism database (dbSNP, http://www.ncbi.nlm.nih.gov/projects/SNP/snp_summary.cgi), and the Exome Aggregation Consortium (ExAC, http://exac.broadinstitute.org) were excluded. Next, all genes causing AI were analyzed, such as *AMELX*, *ENAM*, *AMBN*, *KLK4*, *MMP20*, etc. [5, 9, 10, 21]. The pathogenicity of the remaining gene mutations was predicted using Sorting Intolerant From Tolerant (SIFT, http://sift.jcvi.org/) and polymorphism phenotyping (PolyPhen-2, http://genetics.bwh.harvard.edu/pph2/) next.

### Sanger sequencing

The identified mutations in *AMELX* and *ENAM* were validated for their segregation within each family by Sanger sequencing. Using the PrimeSTAR® HS DNA Polymerase (Takara, Tokyo, Japan), polymerase chain reaction (PCR) was performed by using the intron-exon boundaries specific primers as described previously [22, 23], and the products were sent to Rui Biotech (RuiBiotech, Beijing, China) for further purification and Sanger sequencing.

### Minigene splicing assay

The DNA fragments (2427 bp, NG_012040.1: g.9824-g.12,250) including exons 5, 6, partial exon 7 and introns 5, 6 of the AMELX gene were amplified from gDNA of the control using a forward primer AMELX-F with the restriction site *Bam* HI and a reverse primer AMELX-R with the restriction site *Xho*I. The mutant fragments were obtained with mutagenesis primers of AMELX-MT-F and AMELX-MT-R. Similarly, the DNA fragments (2393 bp, NG_013024.1: g.5758-g.8150) including exons 3, 4, partial exon 5 and introns 3, 4 of the ENAM gene were amplified from gDNA of the control using a forward primer ENAM-F with the restriction site *Bam* HI and a reverse primer ENAM-R with the restriction site *Xho* I. The mutant fragments were obtained with mutagenesis primers of ENAM-MT-F and ENAM-MT-R. The sequences of the DNA fragments were shown in supplemental files. The amplified products were cloned into the pMini-CopGFP vectors (HITRO Biotech, Beijing, China) respectively. Human embryonic kidney 293T (HEK293T) cells were cultured in Dulbecco’s modified Eagle’s medium supplement with 10% fetal bovine serum (HyClone) and incubated at 37°C and 5% CO_2_. When the cells were 70-90% confluent, wild-type (WT) and mutant (MT) vectors were transfected into HEK293T cells using Lipofectamine 3000 Reagent (Thermofisher) for splicing assay following instructions. Total RNA was isolated after 36 h, and cDNA was synthesized with the PrimeScript^™^ RT Master Mix (Takara). PCR was performed with primers (AMELX-S-F and AMELX-F-R for the AMELX gene; ENAM-S-F and ENAM-S-R for the ENAM gene). The sequences of the primers used in the splicing assay were shown in Table 1. The PCR products were sent to the Rui Biotech for sanger sequencing.

**Table 1:** Primers used in minigene splicing assay

Primer	Sequence (5’-3’)
AMELX-F	AAGCTTGGTACCGAGCTCGGATCCGTGCTTACCCCTTTGAAGTGGTACCAGA
AMELX-R	TTAAACGGGCCCTCTAGACTCGAGCACTCCTGAAAGCATCTGAAGTATTCATTCC
AMELX-MT-F	GGAGGAAGTGATGAGTATATTTTGAAGCCACTACAATGC
AMELX-MF-R	TACTCATCACTTCCTCCCGCTTGGTCTTGTCT
AMELX-S-F	TACCAGAGCATAAGGCCACC
AMELX-S-R	TCCCCTCTCATCTTCTGATCT
ENAM-F	AAGCTTGGTACCGAGCTCGGATCCATGTTGGTGCTTCGGTGCAGGCTTGGAA
ENAM-R	TTAAACGGGCCCTCTAGACTCGAGCTCCTCACTTTTACTGCTAAATCCAGGC
ENAM-MT-F	CTATGCCAGTGGGTATTTTTTAAATGTTAGCTCTTCTCTTTG
ENAM-MT-R	AATACCCACTGGCATAGCAACAGAATTACCAA
ENAM-S-F	ATGTTGGTGCTTCGGTGC
ENAM-S-R	TAAATCCAGGCATTCGGGG

### Prediction of three-dimensional (3D) structures of the wild-type and mutant protein

To analyze the effect of the splicing mutations on the structures of AMELX and ENAM protein, 3D structures of the wild-type and mutant protein of *AMELX* and *ENAM* were predicted by AlphaFold2 (https://colab.research.google.com/github/sokrypton/ColabFold/blob/main/AlphaFold2.ipynb). The results were visualized by RCSB PDB (https://www.rcsb.org/3d-view). Conservation analysis of ENAM in different species was performed using ClustalW2 (http://www.ebi.ac.uk/tools/clustalw2).

## Results

### Family 1

**Fig. 1:**
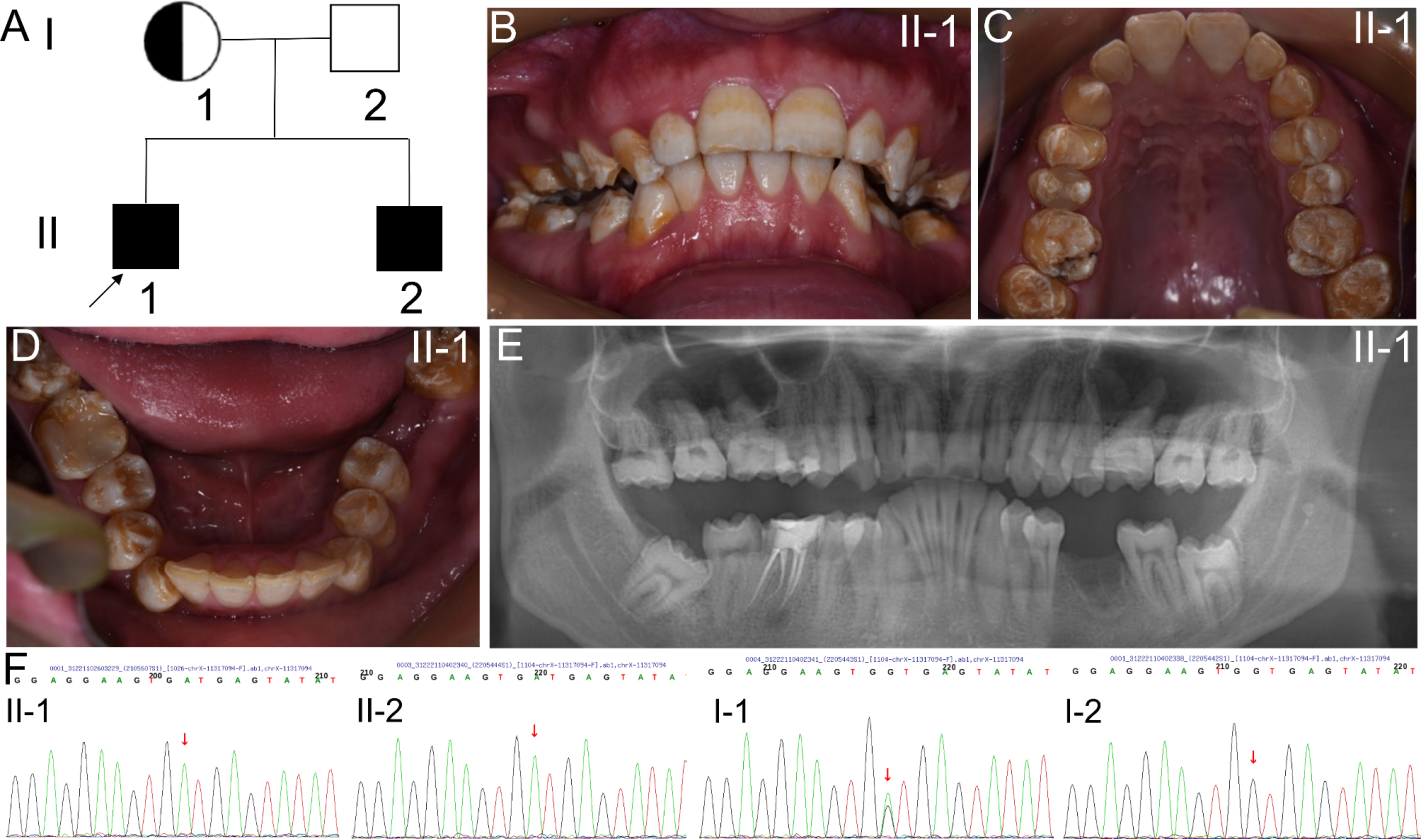
Family 1. **A** Pedigree tree of family 1. Symbols filled with black designate affected individuals, and half blacked represents the hemizygote. The black arrow indicates the proband. **B-D** Intraoral photographs of the proband (II-I). The enamel is thin, rough, and stained. The molar cusps exhibit malformation and hypomineralization. **E** Panoramic photograph of II-1. The enamel is thin, even in the unerupted third molar. **F** Sanger sequencing. The The proband (II-1) and his younger brother (II-2) were hemizygotes, while his mother was a heterozygote (I-1). The mutation could not be detected in his father (I-2). Red arrows indicated the mutation site

The proband was a 26-year-old man from a nonconsanguineous Chinese family (II-1, Fig. 1A). Clinical examination revealed that the enamel of the proband was thin, rough, and stained. Varying degrees of enamel defect were also observed in posterior teeth. The molar cusps exhibited malformation and hypomineralzation, though the patient is not specifically susceptible to dental caries (Fig. 1B-D). The panoramic radiograph clearly showed enamel thinness, but rather normal mineralized contrast with dentin (Fig. 1E). The characteristic features of hypolastic and hypomaturation AI (type IE, OMIM: 301,200) were identified. His younger brother presented similar clinical features, and his mother, the heterozygote, exhibited normal dentition. No participants have exhibited any signs of syndromic diseases, such as osteoporosis, sparse hair and hearing loss, etc.

**Fig. 2:**
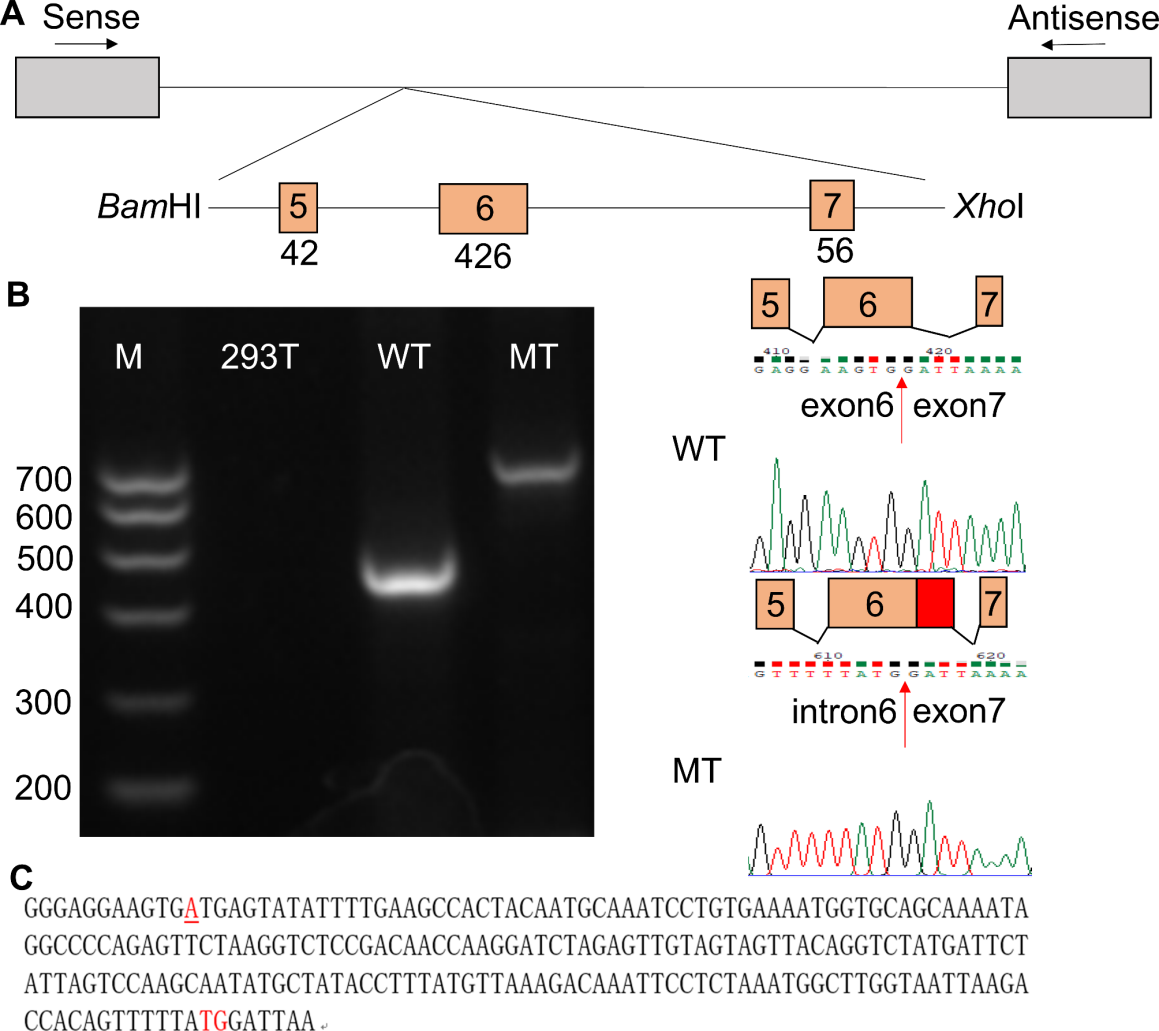
Minigene splicing assay and sequencing of *AMELX*. **A** Diagram of minigene cloning. After double digestion of *Bam*HI and *Xho*I restriction endonucleases, a genomic fragment including exons 5, 6, and partial exon 7 of *AMELX* was cloned into pMini-CopGFP vector. Boxes represent exons and horizontal lines represent introns. The length of the exon is shown under the boxes. **B** Splicing assay. Box filled with red represents the retained intron. Electrophoresis result revealed that the wild type (WT) showed a smaller band with exon 5, 6, and partial exon 7, while the mutant (MT) showed a bigger size **but weaker** band including partial intron 6 additionally. Sequencing chromatograms are shown on the right side. M: marker. **C** Nucleotide sequence of partial retained *AMELX* intron 6. The red underline represents the mutation site, and the red “ATG” is the translation start site. The red “TG” ended the remained intron 6

WES of the proband revealed a previously undescribed splicing mutation of *AMELX* (NM_001142.2: c.570 + 1G > A), which was located in intron 6. The identified mutation was verified subsequently by Sanger sequencing (Fig. 1F). The mutation was also detected in the family members (Fig. 1F).This variant is not listed in the human gene mutation database (HGMD, http://www.hgmd.cf.ac.uk/ac/index.php) and in the dbSNP database. SPIDEX predicted it to affect pre-mRNA splicing (http://tools.genes.toronto.edu). This mutation was predicted to be disease-causing by Mutation Taster (http://www.mutationtaster.org). According to ACMG (American College of Medical Genetics and Genomics) guidelines, this mutation was predicted to be P (pathogenic). The alternative splicing isoforms *AMELX* usually skip exon 4 during pre-mRNA splicing. A silent mutation in exon 4 results in inclusion of exon 4 [10, 14]. In our study, an in vitro splicing assay showed a strong single amplicon in the wild-type construct included exons 5, 6, and partial exon 7. However, there was a weak but bigger size amplicons in mutant construct (Fig. 2A, B, Supplemental Fig. 1). The mutant construct included partial intron 6 additionally (5’ 210 bp, Fig. 2C). In summary, the splicing mutation of *AMELX* led to partial retention of intron 6.

**Fig. 3:**
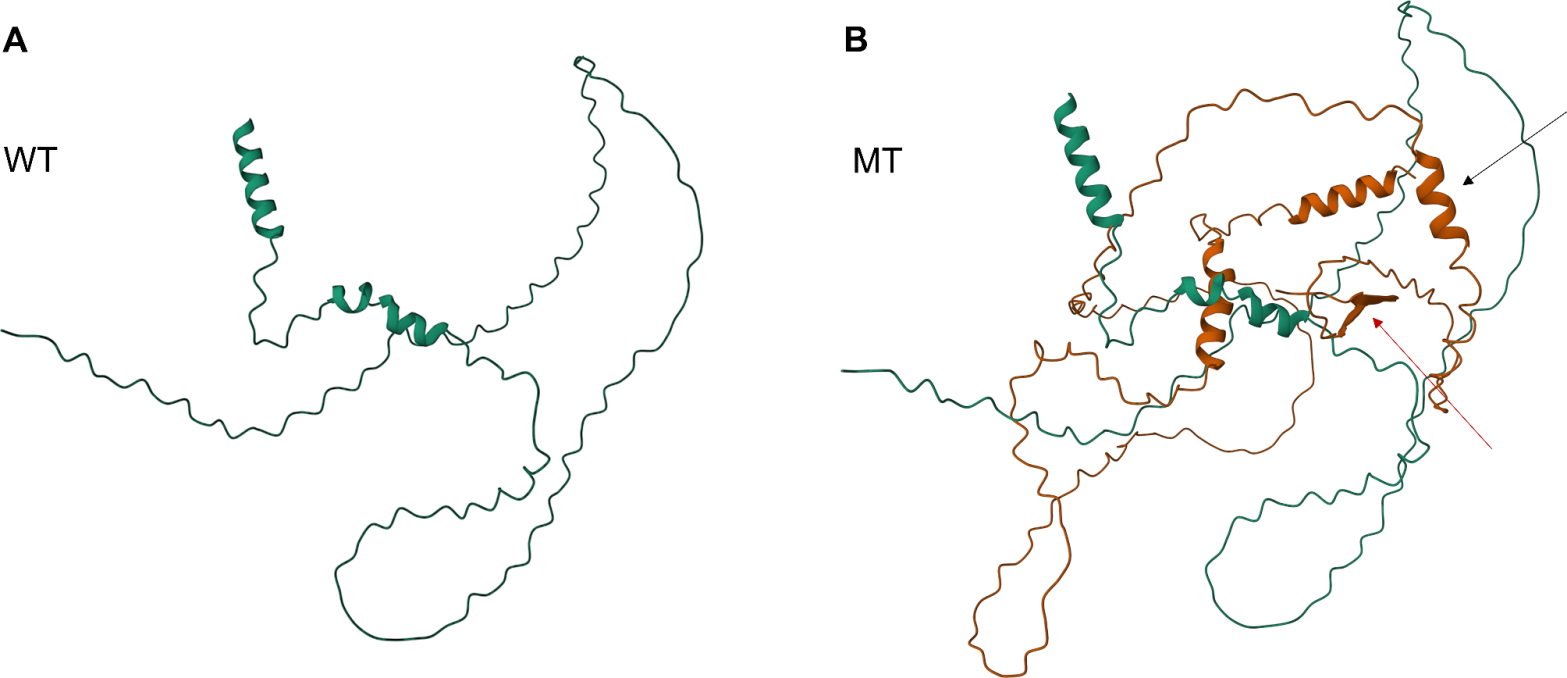
The 3D structure of AMELX predicted by AlphaFold2. **A** Wild type (WT); **B** Mutant (MT). The black arrow indicates the α helix, and the red arrow indicates the β strand

The 3D structures of wild type and mutant AMELX were predicted by AlphaFold2. The predicted structures showed that there were obvious difference in protein folding between the mutation and normal control. Meanwhile, the secondary structure was significantly changed. The α helix and β strand were increased, based on the models for both wild-type and the mutation proteins (Fig. 3).

### Family 2

**Fig. 4:**
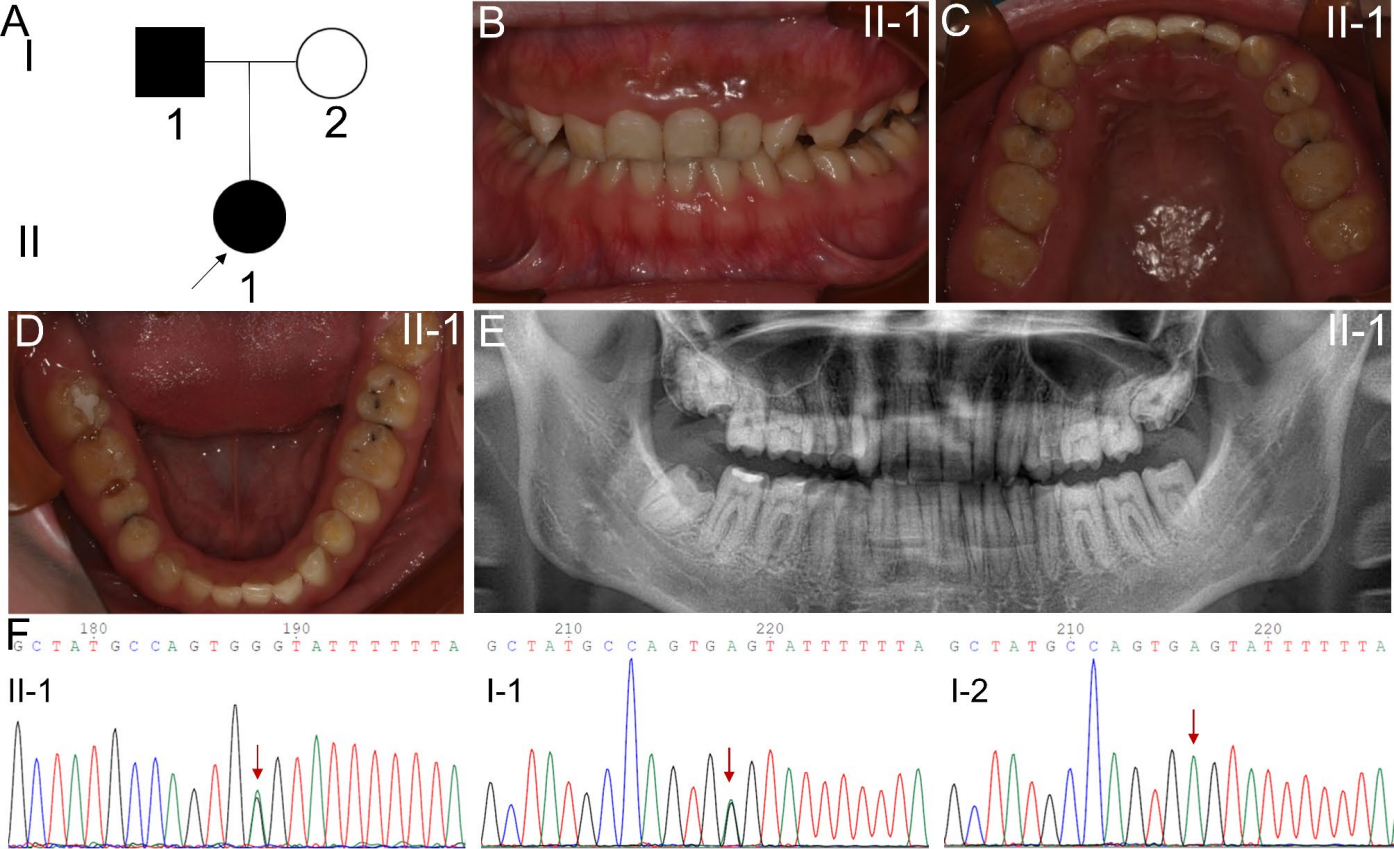
Family 2. **A** Pedigree tree of family 1. The black arrow indicates the proband. **B-D** Intraoral photographs of the proband (II-I). The enamel is thin, while the texture of enamel was normal. Intact cusps and normal adjacent space could not be observed. **E** Panoramic photograph of II-1. The enamel is normal mineralized with normal contrast of enamel to dentin. **F** Sanger sequencing. The proband (II-1) and her father (I-1) were heterozygotes, while this variant could not be detected in her mother (I-2). The site of the splicing mutation was indicated by red arrow

The proband in family 2 was a 23-year-old woman from a nonconsanguineous Chinese family, presenting a typical pitted hypoplastic AI phenotype (type IB, OMIM: 104,500, Fig. 4A). She had irregularly enamel with reduced thickness in general, and the texture of enamel was normal. Furthermore, intact cusps and normal adjacent space could not be observed, dental caries were detected in posterior teeth (Fig. 4B-D). Her father was observed to have a similar dental phenotype and her mother was not affected. The panoramic radiograph of the proband showed that there was a thin layer of enamel covering normal developed dentin, especially in the posterior molars area. The hypomaturation feature could not be noticed in the panoramic radiograph based on the normal contrast of enamel to dentin (Fig. 4E). Syndromic diseases, such as osteogenesis imperfecta, could not be observed in all family members.

**Fig. 5:**
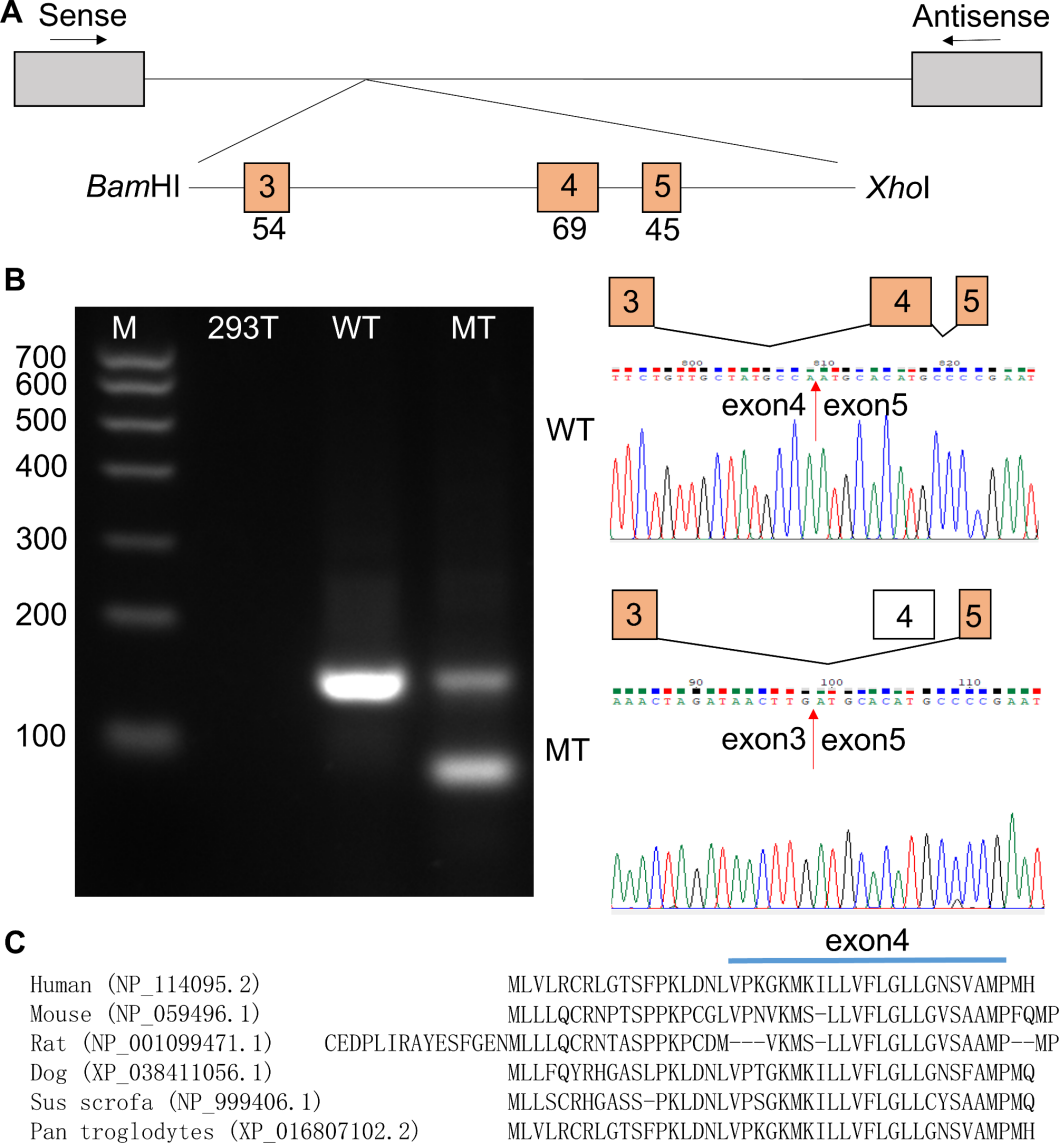
Minigene splicing assay and sequencing of *ENAM*. **A** Diagram of minigene cloning. After double digestion of *Bam*HI and *Xho*I restriction endonucleases, a genomic fragment including exons 3, 4, and partial exon 5 of *ENAM* was cloned into pMini-CopGFP vector. Boxes represent exons and horizontal lines represent introns. The length of the exon is shown under the boxes. **B** Splicing assay. Electrophoresis result revealed that the wild type (WT) showed one band with exons 3, 4, and partial exon 5, while the mutant (MT) showed two bands: a bigger size band (exons 3, 4, and partial exon 5) and a smaller size band (exons 3 and partial exon 5). Sequencing chromatograms are shown on the right side. M: marker. **C** Comparison of ENAM amino acids across different species. The exon 4 was highly conserved

**Fig. 6:**
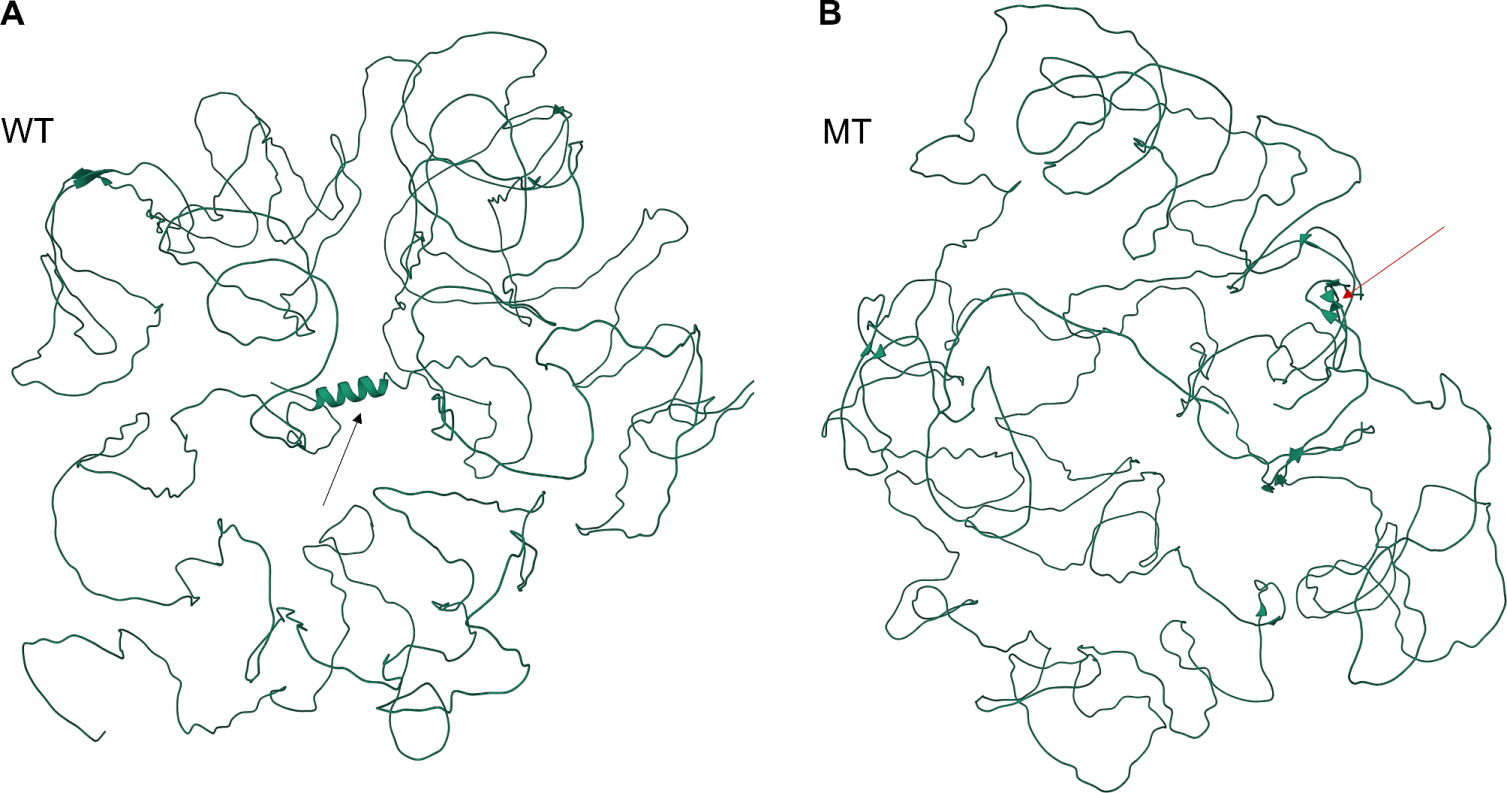
The 3D structure of ENAM predicted by AlphaFold2. **A** Wild type (WT); **B** Mutant (MT). The black arrow indicates the α helix, and the red arrow indicates the β sheet

Genomic analysis of the proband in family 2 revealed a novel splicing mutation (NM_031889.2: c.123 + 4 A > G) in the intron 4 of *ENAM*. Sanger sequencing was also performed to verify this mutation, and her father was a heterozygous mutation (Fig. 4F). This mutation has never been reported before and could not be found in HGMD and dbSNP databases, which was predicted to affect pre-mRNA splicing by SPIDEX. This mutation was predicted to be disease-causing by Mutation Taster and VUS (uncertain significance) according to ACMG guidelines. Minigene splicing assay revealed that there was an obvious difference between the wild-type and mutant vectors. The wild-type construct revealed one band included exons 3, 4, and partial exon 5, and the mutant construct showed two bands: a weaker band include exons 3, 4, and partial exon 5, and a smaller size band without exon 4 (Fig. 5A, B, Supplemental Fig. 2), which indicated that the messenger without exon 4 might reduce the amount of available messenger that contains exons 3, 4, and partial exon 5. The c.123 + 4 A > G mutation led to exon 4 skipping, while the exon 4 was highly conserved (Fig. 5C). Compared with the 3D structure of normal ENAM, the mutant protein was predicted to have more β sheet and less α helix, which vastly affect the secondary structure of the protein (Fig. 6). This variant was predicted to cause a loss of function effect of mutant protein.

## Discussion

In this study, we identified two novel splicing mutations in *AMLEX* and *ENAM* causing hypoplastic/hypomaturation AI and hypoplastic AI, respectively. The intron-exon boundary mutation on *AMLEX* in proband 1 resulted in a partial inclusion of intron 6, which would change the secondary structure of amelogenin. The proband 2 with a typical hypoplastic AI phenotype, mapped the disease-causing mutation to a novel mutation in intron 4 of *ENAM*. Furthermore, the minigene splicing assay revealed that the mutation also influenced mRNA splicing by skipping of exon 4, which was predicted to destruct the signal peptide.

Through selective inclusion or exclusion of exons or introns during pre-mRNA processing, alternative splicing generates distinct mRNA variants and is essential for development, homeostasis, and renewal [24]. Sixteen alternative splicing transcripts can be observed in murine *Amelx* [25, 26]. Splicing mutations can create new splice sites or enhancer sequences, which create aberrant transcripts and contribute to disease [12]. In this study, for the first time, we reported a splicing mutation of *AMELX* gene could cause intron retention. It is well-known that mRNAs with retained introns are generally restricted from exiting the nucleus [27]. Most intron retention in mammalian mRNAs was considered to downregulate gene expression by RUST (regulated unproductive splicing and translation) or NMD (nonsense mediated decay) [28, 29]. As we all know, amelognenin participates in enamel matrix deposition and mineralization [30, 31]. The lack of normal transcripts would prevent the elongation of the crystal, and promotes apoptosis of ameloblasts, leading to severe defects of enamel bio-mineralization [32]. Consistently, decreased enamel thickness and hypomineralization could be observed in the dentitions of proband 1, indicating that the splicing mutation of *AMELX* results in enamel malformation and hypomineralization.

Exon skipping is the most common alternative splicing, which is reported in genetic diseases such as Duchenne muscular dystrophy [24, 33]. Splicing mutations in *ENAM* was predicted to cause intron retention or exon skipping [34, 35]. In our study, the splicing mutation in proband 2 led to exon 4 skipping. Human *ENAM* encodes 1142 amino acids, including a 39-amino-acid length signal peptide encoded by exon 3 and exon 4 [20]. Exon 4 skipping would delete 23 amino acids and interrupt the signal peptide as result. The c.123 + 4 A > G mutation was predicted to alter the secondary structure of enamelin and downregulate the ENAM expression.

Dose-dependent effect can be investigated in the enamel phenotypes caused by *ENAM* mutations. In vitro experiment showed that the secretion of mutant protein caused by a splicing mutation was reduced [20]. When only one allele is mutated, the phenotype may be slight or not obvious, and when both alleles are mutated, the phenotype is significant [9]. Exon skipping was predicted to cause a more severe phenotype [36]. Meanwhile, the proband in family 2, who was a heterozygous mutation, presented a phenotype with generalized thinner enamel thickness and localized enamel pitting, which was consistent with the previous reports. Haploinsufficiency would result in hypoplastic enamel.

In summary, we characterized two novel splicing mutations in *AMELX* and *ENAM* in two Chinese pedigrees. The splicing mutation in *AMELX* caused the partial retention of intron 6 during pre-mRNA splicing. The mutation in intron 4 of *ENAM* resulted in exon 4 skipping. Splicing mutations in *AMELX* and *ENAM* lead to AI. These results expand the spectrum of gene mutations causing amelogenesis imperfecta, and broaden our understanding of pathologic mechanisms of splicing mutations causing AI.

### Electronic supplementary material

Below is the link to the electronic supplementary material.

Supplementary Material 1

Supplementary Material 2

Supplementary Material 3

## Data Availability

The datasets generated and/or analysed during the current study are available in the Sequence Read Archive (SRA) repository (PRJNA997257).

## References

[1] Lacruz RS, Habelitz S, Wright JT, Paine ML (2017). Dental enamel formation and implications for oral health and disease. Physiol Rev.

[2] Gil-Bona A, Bidlack FB (2020). Tooth enamel and its dynamic protein matrix. Int J Mol Sci.

[3] Balic A, Thesleff I. Tissue interactions regulating tooth development and Renewal. 1st ed. Elsevier Inc; 2015. 10.1016/bs.ctdb.2015.07.006 26589925

[4] Wald T, Spoutil F, Osickova A (2017). Intrinsically disordered proteins drive enamel formation via an evolutionarily conserved self-assembly motif. Proc Natl Acad Sci U S A.

[5] Smith CEL, Poulter JA, Antanaviciute A (2017). Amelogenesis imperfecta; genes, proteins, and pathways. Front Physiol.

[6] Crawford PJM, Aldred M, Bloch-Zupan A (2007). Amelogenesis imperfecta. Orphanet J Rare Dis.

[7] Hu JCC, Chun YHP, Al Hazzazzi T, Simmer JP (2007). Enamel formation and amelogenesis imperfecta. Cells Tissues Organs.

[8] Aldred MJ, Savarirayan R, Crawford PJM (2003). Amelogenesis imperfecta: a classification and catalogue for the 21st century. Oral Dis.

[9] Yu S, Zhang C, Zhu C (2022). A novel ENAM mutation causes hypoplastic amelogenesis imperfecta. Oral Dis.

[10] Kim YJ, Kang J, Seymen F (2020). Alteration of exon definition causes Amelogenesis Imperfecta. J Dent Res.

[11] Stephanopoulos G, Garefalaki ME, Lyroudia K (2005). Genes and related proteins involved in amelogenesis imperfecta. J Dent Res.

[12] Montes M, Sanford BL, Comiskey DF, Chandler DS (2019). RNA splicing and disease: animal models to therapies. Trends Genet.

[13] Urzúa B, Ortega-Pinto A, Adorno-Farias D (2021). Exploring the Pool of pathogenic variants of Amelogenesis Imperfecta: an Approach to the understanding of its Genetic Architecture. Front Dent Med.

[14] Cho ES, Kim KJ, Lee KE (2014). Alteration of conserved alternative splicing in AMELX causes enamel defects. J Dent Res.

[15] Duan X, Yang S, Zhang H (2019). A Novel AMELX Mutation, its phenotypic features, and Skewed X inactivation. J Dent Res.

[16] Leban T, Trebušak Podkrajšek K, Kovač J, et al. An Intron c.103-3T > C variant of the AMELX Gene causes combined Hypomineralized and hypoplastic type of Amelogenesis Imperfecta: Case Series and Review of the literature. Genes (Basel). 2022;13. 10.3390/genes13071272. 10.3390/genes13071272 PMC9321068 35886055

[17] Kim YJ, Kim YJ, Kang J (2017). A novel AMELX mutation causes hypoplastic amelogenesis imperfecta. Arch Oral Biol.

[18] Hu JCC, Yamakoshi Y (2003). Enamelin and autosomal-dominant amelogenesis imperfecta. Crit Rev Oral Biol Med.

[19] Hu JCC, Hu Y, Smith CE (2008). Enamel defects and ameloblast-specific expression in Enam knock-out/lacZ knock-in mice. J Biol Chem.

[20] Kim YJ, Lee Y, Zhang H (2021). Translational attenuation by an intron retention in the 5′ utr of enam causes amelogenesis imperfecta. Biomedicines.

[21] Kim YJ, Zhang H, Lee Y, et al. Novel WDR72 mutations causing Hypomaturation Amelogenesis Imperfecta. J Pers Med. 2023;13. 10.3390/jpm13020326. 10.3390/jpm13020326 PMC9965932 36836560

[22] Chan HC, Mai L, Oikonomopoulou A (2010). Altered enamelin phosphorylation site causes amelogenesis imperfecta. J Dent Res.

[23] Kim J, Simmer JP, Hu YY (2004). Amelogenin p: M1T and p.M1T and p.W4S mutations underlying hypoplastic X- linked amelogenesis imperfecta. J Dent Res.

[24] Verhaart IEC, Aartsma-Rus A (2019). Therapeutic developments for Duchenne muscular dystrophy. Nat Rev Neurol.

[25] Bartlett JD, Ball RL, Kawai T (2006). Origin, splicing, and expression of rodent amelogenin exon 8. J Dent Res.

[26] Veis A (2003). Amelogenin gene splice products: potential signaling molecules. Cell Mol Life Sci.

[27] Grabski DF, Broseus L, Kumari B (2021). Intron retention and its impact on gene expression and protein diversity: a review and a practical guide. Wiley Interdiscip Rev RNA.

[28] Wong JJL, Schmitz U (2022). Intron retention: importance, challenges, and opportunities. Trends Genet.

[29] Green RE, Lewis BP, Hillman RT (2003). Widespread predicted nonsense-mediatedmRNA decayof alternatively-spliced transcripts of human normal and disease genes. Bioinformatics.

[30] Gibson CW, Kulkarni AB, Wright JT (2005). The use of animal models to explore amelogenin variants in amelogenesis imperfecta. Cells Tissues Organs.

[31] Gibson CW, Yuan ZA, Li Y (2006). Transgenic mice that Express Normal and Mutated Amelogenins. J Dent Res.

[32] Barron MJ, Brookes SJ, Kirkham J (2010). A mutation in the mouse Amelx tri-tyrosyl domain results in impaired secretion of amelogenin and phenocopies human X-linked amelogenesis imperfecta. Hum Mol Genet.

[33] Keren H, Lev-maor G, Ast G (2010). Alternative splicing and evolution: diversification, exon definition and function. Nat Publ Gr.

[34] Rajpar MH, Harley K, Laing C (2001). Mutation of the gene encoding the enamel-specific protein, enamelin, causes autosomal-dominant amelogenesis imperfecta. Hum Mol Genet.

[35] Kim J, Seymen F, Lin BP (2005). ENAM mutations in autosomal-dominant amelogenesis imperfecta. J Dent Res.

[36] Hart PS, Michalec MD, Seow WK (2003). Identification of the enamelin (g. 8344delG) mutation in a new kindred and presentation of a standardized ENAM nomenclature. Arch Oral Biol.

