# Supplementary figures and images for: Splicing mutations in AMELX and ENAM cause amelogenesis imperfecta
Source: BMC Oral Health. 2023 Nov 20;23:893. doi: 10.1186/s12903-023-03508-8 (PMC10662561; doi:10.1186/s12903-023-03508-8)

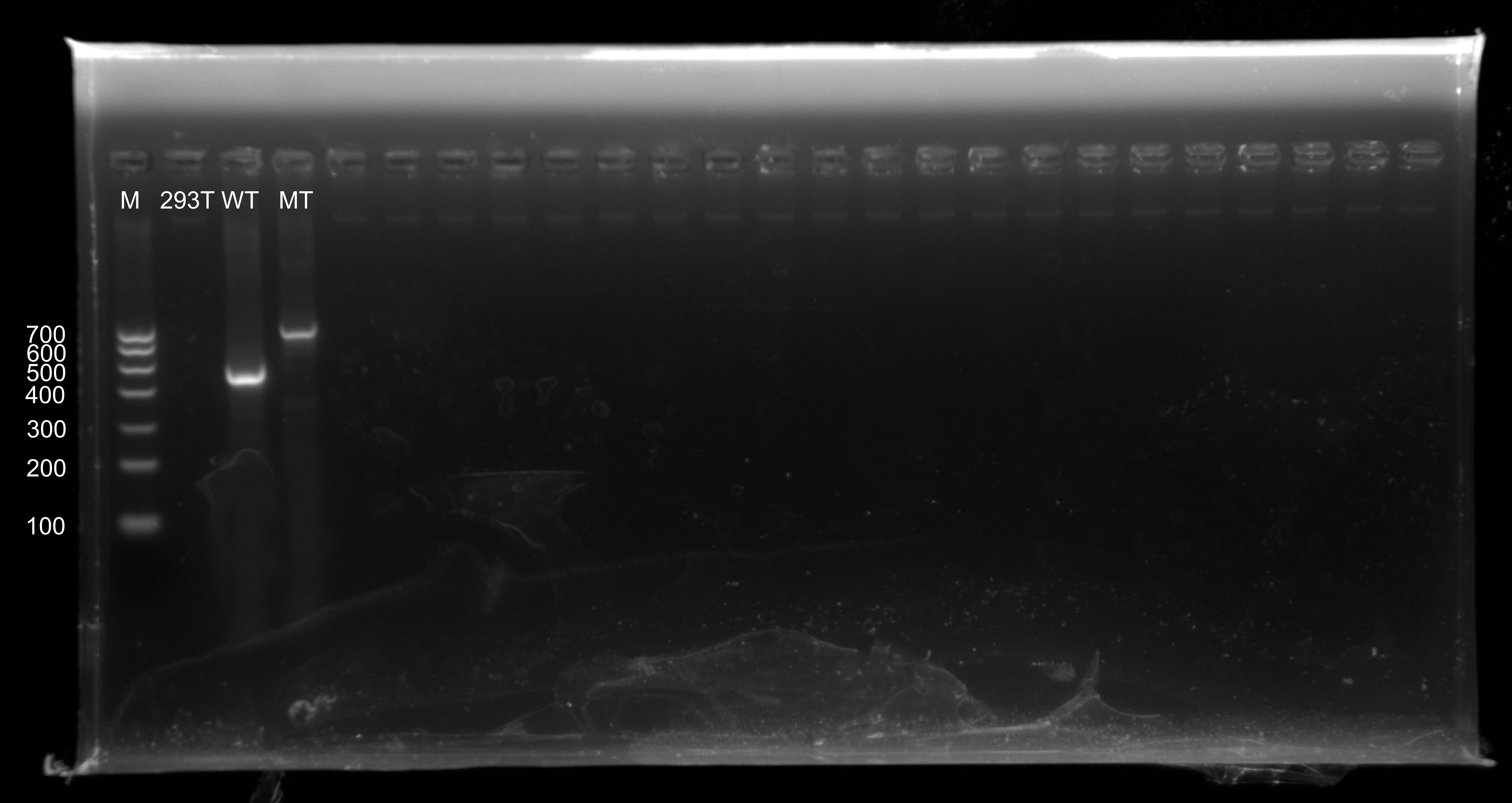

Supplement: Supplementary file 1 — Supplementary Material 1 [file 12903_2023_3508_MOESM1_ESM.png]

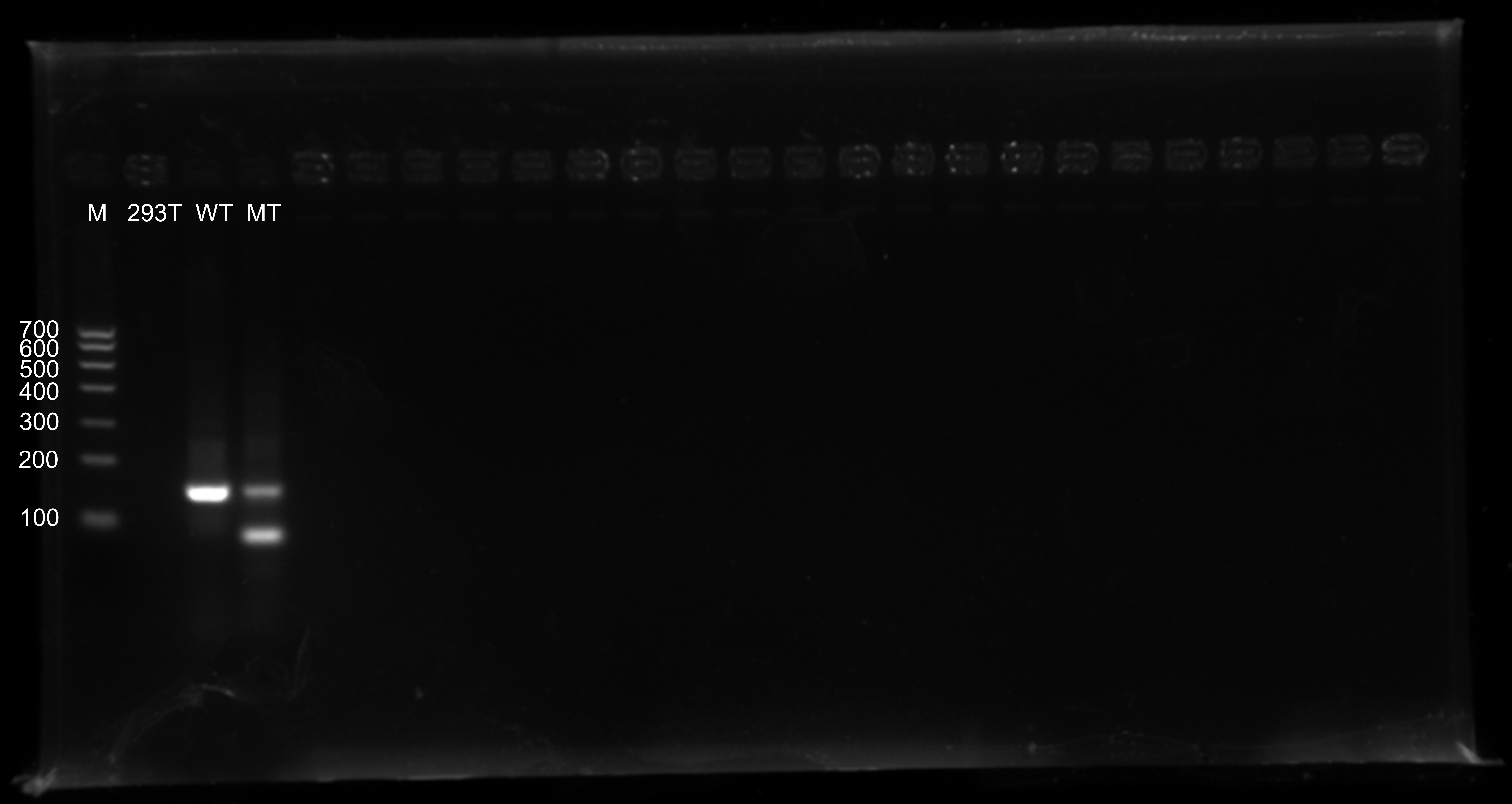

Supplement: Supplementary file 2 — Supplementary Material 2 [file 12903_2023_3508_MOESM2_ESM.png]
