# Supplementary material for: Splicing mutations in AMELX and ENAM cause amelogenesis imperfecta
Source: BMC Oral Health. 2023 Nov 20;23:893. doi: 10.1186/s12903-023-03508-8 (PMC10662561; doi:10.1186/s12903-023-03508-8)
Supplement: Supplementary file 3 — Supplementary Material 3 [file 12903_2023_3508_MOESM3_ESM.docx]

Supplemental Figure legends

Supplemental Figure 1 Gel image of Figure 2B. M: marker. WT: wild type. MT: mutant. Compared with the wild type, the mutant construct showed a bigger size band.

Supplemental Figure 2 Gel image of Figure 5B. M: marker. WT: wild type. MT: mutant. Compared with the wild type, the mutant construct showed an additional smaller size band.

Supplemental table 1 Candidate genes of family 1

| Gene | Location | Diseases |
| --- | --- | --- |
| AMELX (NM_001142.2) | c.570+1G>A | Amelogenesis imperfecta |
| IGF2R (NM_000876.2) | c.4655T>C | Autism spectrum disorder |
| AHNAK (NM_001346445.1) | c.10535T>C | Congenital heart disease |
| ZNF469 (NM_001127464.1) | c.7937C>T | Keratoconus |
| PYGM (NM_001164716.1) | c.1540C>T | McArdle disease |
| BMPR2 (NM_001204.6) | c.2618G>A | Pulmonary arterial hypertension |
| B3GALTL (NM_194318.3) | c.268C>T | Peters Plus syndrome |

Supplemental table 2 Candidate genes of family 2

| Gene | Location | Diseases |
| --- | --- | --- |
| ENAM (NM_031889.2) | c.123+4A>G | Amelogenesis imperfecta |
| PTCH2 (NM_001166292.1) | c.2033C>T | Gorlin syndrome |
| COL17A1 (NM_000494.3) | c.964G>A | Epidermolysis bullosa |
| DHCR7 (NM_001163817.1) | c.1406G>A | Smith-Lemli-Opitz syndrome |
| TP63 (NM_001114980.1) | c.1658G>A | EEC syndrome; AEC syndrome |
| NSD1 (NM_022455.4) | c.4498-295G>A | Sotos syndrome |
| LIFR (NM_001127671.1) | c.258G>T | Stuve-Wiedemann syndrome |

Sequences of the DNA fragments of the AMELX gene (The yellow base represents the mutation site).

WT (NG_012040.1: g.9824-g.12250, 2427bp):

gtgcttacccctttgaagtggtaccagagcataaggccaccggtatgtagacattttgttccttattccctgaaaatattaggcatgcattaaaattcccatattaagtgaaatatcatgtctactccacatgcagacattaatgggaaatttagtttgtaaaaaatcatatctgtgtacacagttacaaatttttgcaaaggaaaaatgaataaaatattcctatagccataatggcaaagaaaacactgctgcttctctggttggagtcacctgagccaatggtaaacctgcctctctgtttctcaccagtacccttcctatggttacgagcccatgggtggatggctgcaccaccaaatcatccccgtgctgtcccaacagcaccccccgactcacaccctgcagcctcatcaccacatcccagtggtgccagctcagcagcccgtgatcccccagcaaccaatgatgcccgttcctggccaacactccatgactccaatccaacaccaccagccaaacctccctccgcccgcccagcagccctaccagccccagcctgttcagccacagcctcaccagcccatgcagccccagccacctgtgcaccccatgcagcccctgccgccacagccacctctgcctccgatgttccccatgcagcccctgcctcccatgcttcctgatctgactctggaagcttggccatcaacagacaagaccaagcgggaggaagtggtgagtatattttgaagccactacaatgcaaatcctgtgaaaatggtgcagcaaaataggccccagagttctaaggtctccgacaaccaaggatctagagttgtagtagttacaggtctatgattctattagtccaagcaatatgctatacctttatgttaaagacaaattcctctaaatggcttggtaattaagaccacagtttttatggtaggtttcaattttactattactgaatttctaccagaatatgtattaccaaaacccattaatagaaatatatattactaaaccccatgaattttaagggcaacagtataagggaatatcagttctccttatatttcaaaggtttgactagcaagaataggctagagttgcactgaaggcttaagacaagagggagcggataattttgagagtgcaaatatctgaacaggctacaaaaggtagacgggaaatctcttcaaaaacctacaggaagattccccatttccagtagttttcaatctaacttggaggcggctaaactaaacatactgttagattccttttctgtactggggttctatagatgattaagcttttagcaagaagttactgcaatttagcactaaatcttccattacaggtagctcttacaaatgaatgggaatagtcaacaaaacaaacttaatctacatccataaagtcttacttctatgtatacgagattatgtgatcctatcatgtatatgtatccaactgtaattccaatttatacatgttattgatgatttgctactgagaagaagagagaagtgaggtggaaatgaccaggataagaagccaggacacaaaggttccaattctggctttgccctcaaagacgaggcagtattgtaaaagttacttcaaatgtatcggtgttttattttcttttaaatgggggaaaatgacgaaatcagattattttcaagtctctgtccaattataaataccatagtttctgaatttaaaaaaatcataatatatgtcataaatggcttcataattgtgagcatgtttacggaaaatatggggcagaattttttgaaaattgattgaatcccaagtaatcggtgcctatcattggctattctagtccaaggcacatgttctctctgtacatagaaaatgcatttacttctttatgaataattaataccatgaactttataatgtgctcacatcttgacaaagctatttatggaaaggtgactttgggcagatagtttgaactctttaaaactcagtttctttttgtgtaaaatttgagtataaacattgatagtttcttagagttgttttatggaaaacaaaatagcgtgataagcttggagcctgacatgcaagacgtaccccccaaaaaggtagcaattgttattttattataaaataataggctttaagtgtcctgaaggtggaagcagtcctcatggacacctaatatctaatgacaacgaaataacaaagaaacttcagaaattatagagttcaacttaaatggttgtacattgttttgacaaaactgaagccagacatgttattgtaaatggtactcactaggaacatttgtaaattattttaactgttcttttgcaatttttttcaggattaaaagatcagaagatgagaggggaatgaatacttcagatgctttcaggagtg

MT:

gtgcttacccctttgaagtggtaccagagcataaggccaccggtatgtagacattttgttccttattccctgaaaatattaggcatgcattaaaattcccatattaagtgaaatatcatgtctactccacatgcagacattaatgggaaatttagtttgtaaaaaatcatatctgtgtacacagttacaaatttttgcaaaggaaaaatgaataaaatattcctatagccataatggcaaagaaaacactgctgcttctctggttggagtcacctgagccaatggtaaacctgcctctctgtttctcaccagtacccttcctatggttacgagcccatgggtggatggctgcaccaccaaatcatccccgtgctgtcccaacagcaccccccgactcacaccctgcagcctcatcaccacatcccagtggtgccagctcagcagcccgtgatcccccagcaaccaatgatgcccgttcctggccaacactccatgactccaatccaacaccaccagccaaacctccctccgcccgcccagcagccctaccagccccagcctgttcagccacagcctcaccagcccatgcagccccagccacctgtgcaccccatgcagcccctgccgccacagccacctctgcctccgatgttccccatgcagcccctgcctcccatgcttcctgatctgactctggaagcttggccatcaacagacaagaccaagcgggaggaagtgAtgagtatattttgaagccactacaatgcaaatcctgtgaaaatggtgcagcaaaataggccccagagttctaaggtctccgacaaccaaggatctagagttgtagtagttacaggtctatgattctattagtccaagcaatatgctatacctttatgttaaagacaaattcctctaaatggcttggtaattaagaccacagtttttatggtaggtttcaattttactattactgaatttctaccagaatatgtattaccaaaacccattaatagaaatatatattactaaaccccatgaattttaagggcaacagtataagggaatatcagttctccttatatttcaaaggtttgactagcaagaataggctagagttgcactgaaggcttaagacaagagggagcggataattttgagagtgcaaatatctgaacaggctacaaaaggtagacgggaaatctcttcaaaaacctacaggaagattccccatttccagtagttttcaatctaacttggaggcggctaaactaaacatactgttagattccttttctgtactggggttctatagatgattaagcttttagcaagaagttactgcaatttagcactaaatcttccattacaggtagctcttacaaatgaatgggaatagtcaacaaaacaaacttaatctacatccataaagtcttacttctatgtatacgagattatgtgatcctatcatgtatatgtatccaactgtaattccaatttatacatgttattgatgatttgctactgagaagaagagagaagtgaggtggaaatgaccaggataagaagccaggacacaaaggttccaattctggctttgccctcaaagacgaggcagtattgtaaaagttacttcaaatgtatcggtgttttattttcttttaaatgggggaaaatgacgaaatcagattattttcaagtctctgtccaattataaataccatagtttctgaatttaaaaaaatcataatatatgtcataaatggcttcataattgtgagcatgtttacggaaaatatggggcagaattttttgaaaattgattgaatcccaagtaatcggtgcctatcattggctattctagtccaaggcacatgttctctctgtacatagaaaatgcatttacttctttatgaataattaataccatgaactttataatgtgctcacatcttgacaaagctatttatggaaaggtgactttgggcagatagtttgaactctttaaaactcagtttctttttgtgtaaaatttgagtataaacattgatagtttcttagagttgttttatggaaaacaaaatagcgtgataagcttggagcctgacatgcaagacgtaccccccaaaaaggtagcaattgttattttattataaaataataggctttaagtgtcctgaaggtggaagcagtcctcatggacacctaatatctaatgacaacgaaataacaaagaaacttcagaaattatagagttcaacttaaatggttgtacattgttttgacaaaactgaagccagacatgttattgtaaatggtactcactaggaacatttgtaaattattttaactgttcttttgcaatttttttcaggattaaaagatcagaagatgagaggggaatgaatacttcagatgctttcaggagtg

Sequences of the DNA fragments of the ENAM gene (The yellow base represents the mutation site).

WT (NG_013024.1: g.5758-g.8150, 2393 bp):

atgttggtgcttcggtgcaggcttggaacctcttttcctaaactagataacttggtgagtactttcatttatttttgccaatacatacaggttctcaaactagatactgtcagaaatacacttgagagggccaaacactactgaaatgtatcagaagactcaaagagcatctgctttcacagatggaatggaagaagaacttacacagtcattgtactttgtggcaatcattatgagtgttactttcaactcaaaaaataactaattatagactttaatcactaacttttagagatggaagggatctcagtcatccaacataatatattaattttacaaaagagaaaatgagttttctatttgcaaatataaaggctacatatattatcaagaatcactgtgacagctactagagagatgagatgaatgattaagcaaggcagaaattaaccatccaaaagcacccttaaatcttgggccataatcttagaaacatttagcaaagaaagctttcaccagtattctctttattagtttccacatattgttgtaacctatactaaacactctaaaataacattcagctgttattttcattctctatgatgttctaactctgaaaatctttaaatagtaggaaaggtatttacatactactgcttcctttgtttaattacattctgccaatttttctaatgtatgaaagataactgattacacccacaatttaggaaaatgtccattataaaaatgctaaagcgtcatttatgttgctcctataggaagtaaacagaagggtgagcattggctccaattatgctagatagattttatgtccagaaaaggaagaaagaatgaaaggaatgaaaacaactgagtgtctacatgttggttactctgctaggaaatggactatatctgatatacgatgaaaatgaatcattctgattaaaaccttttattttcctctattgtatatgcaaatagaaagttttcttaagaaattactcaagtaaatgtttcagaataaaccttgttccaagatgtaaatacgagacttaattttacaagtctttcataattaactagagtgggataaatgaatgaaccccgttaaaaacaatctccaaacttcatacataagtctatatgtttaatcaaggtatctaataaagagataaatttactattctgcctatttcctatttcttcctattttcctgaccatcttagagcataagtgatttttttttttttttgagacagagtctcactctgtcgcccaggctggagtgcaatggcacgatcttggctcactgcaacctcctcctcctgggttcaagcgattctcctgcctcagcctcccaagtggctggtattacaggtgtgagacaccacacccggctaatttttgtatttttagtagagacaaggtttcaccatgttggctcagctggtctcaagctcctgtcctcaggggatccacccacctcagcctcccaaagtgatgggattaaggcgtgagccaccgcgcctggccacataagtgatttttaaaggaagtgatgatgatgataataataataacaggaacaatagaggaaaccaggaaaaaaataagattctgcatattccagtttacttagtctctatataacagaatattatctataacatcttagaaagtagcacaaagttttgattttggagtgtgccctccttgaaactgagattattctgtacaatgaatgtagttggacatcactatttctttataaatcaactagtaactagaaactcaaacccattaatattcctatttgggggataacctcctaatttctaattttcacattctggtgctatgatatctctctctttctctctctctctgtctctctctctttccctgtctctctctttccctgtctctctctctcacgcacacacacttatatttccttttaatctgactcttttggcagcttgaaaagtaccagatgataactatctaatagtttcttgacataaaataaaactggcagcaggggccccatccatttccatactctccttgacagacaagtaggctagtacttagataagtgcagagtgccctaagcatacttatttcacagaccaaaaataaaaatcaattttttattctaggtaccaaaaggcaaaatgaagattctcctggtctttctagggcttcttggtaattctgttgctatgccagtgagtattttttaaatgttagctcttctctttgtgttccgttaggaacaattgttgagcttatttcaactgatgttctgcatttgtcactgacattctcttacttccagatgcacatgccccgaatgcctggatttagcagtaaaagtgaggag

MT:

atgttggtgcttcggtgcaggcttggaacctcttttcctaaactagataacttggtgagtactttcatttatttttgccaatacatacaggttctcaaactagatactgtcagaaatacacttgagagggccaaacactactgaaatgtatcagaagactcaaagagcatctgctttcacagatggaatggaagaagaacttacacagtcattgtactttgtggcaatcattatgagtgttactttcaactcaaaaaataactaattatagactttaatcactaacttttagagatggaagggatctcagtcatccaacataatatattaattttacaaaagagaaaatgagttttctatttgcaaatataaaggctacatatattatcaagaatcactgtgacagctactagagagatgagatgaatgattaagcaaggcagaaattaaccatccaaaagcacccttaaatcttgggccataatcttagaaacatttagcaaagaaagctttcaccagtattctctttattagtttccacatattgttgtaacctatactaaacactctaaaataacattcagctgttattttcattctctatgatgttctaactctgaaaatctttaaatagtaggaaaggtatttacatactactgcttcctttgtttaattacattctgccaatttttctaatgtatgaaagataactgattacacccacaatttaggaaaatgtccattataaaaatgctaaagcgtcatttatgttgctcctataggaagtaaacagaagggtgagcattggctccaattatgctagatagattttatgtccagaaaaggaagaaagaatgaaaggaatgaaaacaactgagtgtctacatgttggttactctgctaggaaatggactatatctgatatacgatgaaaatgaatcattctgattaaaaccttttattttcctctattgtatatgcaaatagaaagttttcttaagaaattactcaagtaaatgtttcagaataaaccttgttccaagatgtaaatacgagacttaattttacaagtctttcataattaactagagtgggataaatgaatgaaccccgttaaaaacaatctccaaacttcatacataagtctatatgtttaatcaaggtatctaataaagagataaatttactattctgcctatttcctatttcttcctattttcctgaccatcttagagcataagtgatttttttttttttttgagacagagtctcactctgtcgcccaggctggagtgcaatggcacgatcttggctcactgcaacctcctcctcctgggttcaagcgattctcctgcctcagcctcccaagtggctggtattacaggtgtgagacaccacacccggctaatttttgtatttttagtagagacaaggtttcaccatgttggctcagctggtctcaagctcctgtcctcaggggatccacccacctcagcctcccaaagtgatgggattaaggcgtgagccaccgcgcctggccacataagtgatttttaaaggaagtgatgatgatgataataataataacaggaacaatagaggaaaccaggaaaaaaataagattctgcatattccagtttacttagtctctatataacagaatattatctataacatcttagaaagtagcacaaagttttgattttggagtgtgccctccttgaaactgagattattctgtacaatgaatgtagttggacatcactatttctttataaatcaactagtaactagaaactcaaacccattaatattcctatttgggggataacctcctaatttctaattttcacattctggtgctatgatatctctctctttctctctctctctgtctctctctctttccctgtctctctctttccctgtctctctctctcacgcacacacacttatatttccttttaatctgactcttttggcagcttgaaaagtaccagatgataactatctaatagtttcttgacataaaataaaactggcagcaggggccccatccatttccatactctccttgacagacaagtaggctagtacttagataagtgcagagtgccctaagcatacttatttcacagaccaaaaataaaaatcaattttttattctaggtaccaaaaggcaaaatgaagattctcctggtctttctagggcttcttggtaattctgttgctatgccagtgGgtattttttaaatgttagctcttctctttgtgttccgttaggaacaattgttgagcttatttcaactgatgttctgcatttgtcactgacattctcttacttccagatgcacatgccccgaatgcctggatttagcagtaaaagtgaggag
